# Emergence of different crystal morphologies using the coffee ring effect

**DOI:** 10.1038/s41598-018-30879-8

**Published:** 2018-08-21

**Authors:** Kouki Morinaga, Noriko Oikawa, Rei Kurita

**Affiliations:** Department of Physics, Tokyo Metropolitan University, 1-1 Minamioosawa, Hachioji-shi, Tokyo, 192-0397 Japan

## Abstract

Macroscopic patterns in nature formed during crystal growth e.g. snow crystals have a significant influence on many material properties, such as macroscopic heat conduction, electrical conduction, and mechanical properties, even with the same microscopic crystal structure. Although crystal morphology has been extensively studied in bulk, the formation of patterns induced by re-crystallization during evaporation is still unclear. Here, we find a way to obtain concentric circles, a dendritic pattern, and a lattice pattern by pinning the edge of droplets using the coffee ring effect; only aggregates of crystallites are seen in the absence of pinning. Our systematic study shows that the macroscopic patterns depend both on initial concentration and evaporation rate. In addition, our qualitative analysis suggests that the local concentration of solute at the center of the pattern is related to the macroscopic patterns.

## Introduction

Crystals play an important role in our daily lives. Examples include electronic materials, polymeric materials, and pharmaceuticals^1,2^. The properties of crystals can depend on their microscopic crystal structure, making crystallography the focus of significant scientific and industrial interest. However, crystals also possess a macroscopic morphology^3^. Snow, which has a fractal-like structure, is a typical example which exhibits a wide range of macroscopic patterns^4^. Macroscopic patterns are also closely tied to the dissolution of the crystal, not to mention their electrical and mechanical properties^5–9^. The formation of morphology is strongly influenced by crystal growth, which is itself coupled to the variation of density and concentration in space. Since crystals usually have a high density (with the notable exception of water) or high concentration, there is a notable decrease in the density or concentration at the interface; latent heat is also generated at the interface when the liquid transforms into the crystal. Thus, material transport and heat transport at the crystal interface are also important factors. Due to the coupling between these factors, multiple macroscopic patterns are formed with the same microscopic crystal structure^10–13^.

In everyday life, we are also familiar with the process of *r*e-crystallization e.g. during the evaporation of a droplet of aqueous salt solution, perhaps more so than crystal growth in bulk. Compared to crystal growth in the bulk phase, recrystallization due to evaporation is a much more complex process. For example, evaporation near the edge of a sessile droplet is faster than that on the top of the sessile droplet, since the vapor concentration in air is locally saturated around the droplet^14–18^. The difference in evaporation rate induces flow from the center to the edge. In a similar spirit to studies of evaporating droplets with colloidal particles (coffee ring effect)^19,20^, the evaporation of droplets of water with dissolved salt has been investigated^21–28^. It was reported that a concentric pattern was formed by evaporation of a sessile droplet containing L-ascorbic acid^22^ and bovine serum albumin^23^. Shahidzadeh *et al*. investigated the dynamics and patterns formed using sodium chloride (NaCl) and calcium sulfate (CaSO_4_) solutions for different substrates^25^. For a NaCl solution, they reported that the NaCl crystal nucleates near the edge and is subsequently carried to the center; on the other hand, in a CaSO_4_ solution, the crystallites are observed at the edge of the dried droplet when the substrate is hydrophilic, while a needle-like shape is formed when the substrate is hydrophobic. In addition, it was reported that crystals of sodium sulfate (Na_2_SO_4_) were formed outside the initial contact radius of the droplet^26^. It is thought that the change of surface tension due to crystallization is important. Temperature changes during the crystallization of a sessile droplet were also investigated. In fact, cold spots were found inside droplets^27^. Furthermore, we note that the dynamics of evaporation depends on the concentration^28^. When the concentration of NaCl is over 0.1 M, the evaporation rate decreases due to condensation near the edge. Thus, re-crystallization in a salt solution due to evaporation of water is a complex phenomenon, due to coupling with material transport, the hydrophobicity, thermal behavior, shrinking of the droplet, the change in the surface tension due to crystallization, and the local concentration near the edge. It is clear that further investigation is required.

Here, we investigate the morphology of the crystal formed during evaporation of a sessile droplet of sodium bicarbonate (NaHCO_3_) solution with pinning at the edge. We mix the solution with latex particles in order to pin the edge and stop the droplet shrinking. This method has particular advantages for investigating crystallization by evaporation. For example, the evaporation rate *β*(*t*) becomes constant before the crystallization occurs, as we will describe in detail in the Results section. We perform a series of evaporation experiments while varying the initial concentration and evaporation rate, and find either a concentric circular pattern or a dendritic pattern in the same solution, depending on how these variables are set.

## Results

**Figure 1:**
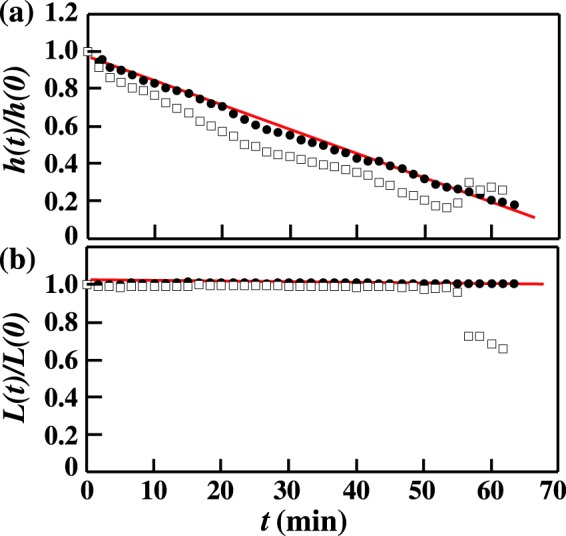
Time evolution of (**a**) the normalized height *h*(*t*) and (**b**) the normalized diameter *L*(*t*) before the crystallization starts. Circles correspond to droplets with the particles, while squares correspond to droplets without particles. When the solution is mixed with particles, *L*(*t*) remains constant due to pinning at the edge due to the coffee ring effect, and *h* decreases linearly before the crystallization starts.

Firstly, we examine the evaporation of a solution droplet mixed with latex particles with a diameter of 0.052 *μ*m. We measure the height *h*(*t*) at the top and the diameter *L*(*t*) of the sessile droplet using top and side views acquired with a video camera. In our experiment, *h*(0) = 1.3 mm and *L*(0) = 5.1 mm for 20 mm^3^ droplets. Figure 1 shows the time evolution of *h*(*t*) and *L*(*t*) of a 4.7 wt% NaHCO_3_ solution droplet at 20 °C and a relative humidity (Rh) of 50%. The filled circles correspond to the time evolution of *h*(*t*) and *L*(*t*) normalized by *h*(0) and *L*(0). We also show *h*(*t*) and *L*(*t*) without the latex particles for reference (squares). We find that *L*(*t*) is unchanged with time until crystallization occurs (*t*_*x*_ = 66 min). This means that the edge of the droplet is pinned by the coffee ring effect. We also find that *h*(*t*) decreases linearly with time. Since *L*(*t*) is constant, the evaporation rate *β*(*t*) is also constant since *β*(*t*)∝ − ∂*h*(*t*)/∂*t*. It is known that *β*(*t*) is proportional to the perimeter of the droplet edge before a critical time^15,16^, while *β*(*t*) drastically increases beyond the critical time^19^; our result is consistent with *β*(*t*) ∝ *L*(*t*). We note that this relation can be observed in all cases which we examined. We also note that the effect of humidity fluctuation (Rh ~ ± 4.0%) is negligible during evaporation. Meanwhile, when the droplet does not contain latex particles, *h*(*t*) suddenly increases and *L*(*t*) spontaneously decreases at *t* = 56 min, before crystallization occurs (*t*_*x*_ = 62 min). From *in-situ* observation of the sessile droplet, we see that the edge of the droplet detaches from the glass and that the droplet is re-shaped at *t* = 56 min. After the crystallization occurs ($t > {t}_{x}$), several additional effects should occur e.g. local temperature change^27^, local density change due to crystallization, local change of surface tension^26^ etc. Thus, the evaporation rate is not expected to be constant after crystallization starts. In our experiments, we were unable to measure the local evaporation rate after $t > {t}_{x}$.

**Figure 2:**
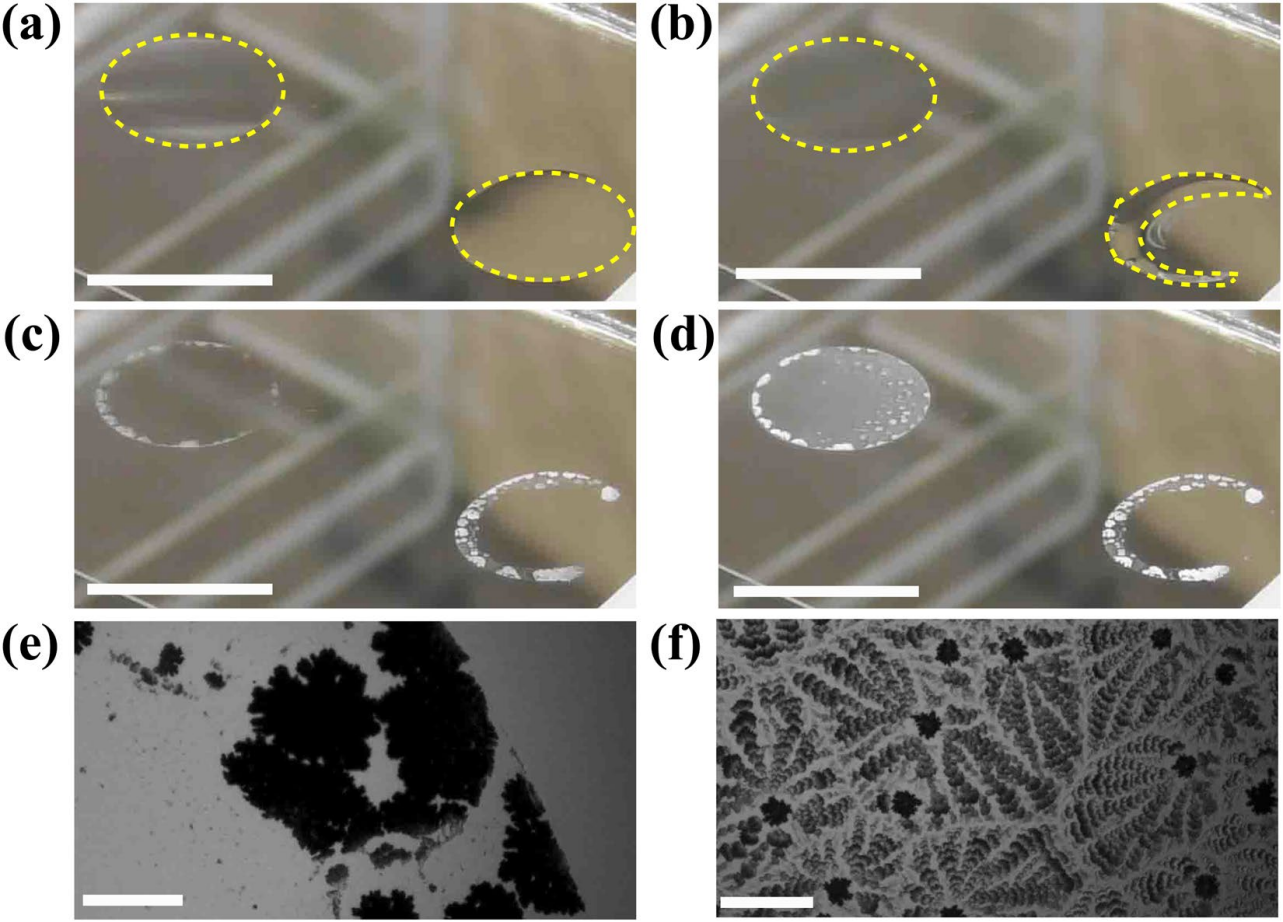
Crystallization dynamics in a sessile droplet at *t* = (**a**) 4665 s, (**b**) 4981 s, (**c**) 5536 s, and (**d**) 5848 s. A left droplet is a 0.47 wt% droplet including the latex particles, on the other hand, a right droplet is a 0.47 wt% droplet without the latex particles. The yellow lines are eye guides for showing edge of the droplet. We find the macroscopic dewetting in the right droplet (**b**). (**e**) The crystal pattern at the initial contact line when the droplet does not contain latex particles. None of the crystals are observed at the center. (**f**) The morphology of the crystal when the droplet contains latex particles. Concentric circles are observed at the center of the dried droplet. Scale bar in (**a**–**d**) corresponds to 5.0 mm and scale bar in (**e**) and (**f**) correspond to 0.1 mm.

Next, we look at crystallization dynamics in a sessile droplet with a pinned edge [See Supplementary video Smovie. 1]. Figure 2(a–d) shows the crystallization dynamics in a 0.47 wt% droplet without the latex particles (right droplet in Fig. 2). We find that the crystallization occurs at the edge first, and that macroscopic dewetting occurs. Finally, the solution is absorbed by the crystal at the edge and the center becomes empty shown as in Fig. 2(e). This is similar to crystallization seen in evaporation from droplets of CaSO_4_ solution^25^. In contrast, a left droplet in Fig. 2(a–d) contains the latex particles. Although the crystallization occurs at the edge first, just like in the droplet without the latex particles, the solution remains at the center due to the strong pinning at the edge. Subsequently, we observe a concentric circular (CC) pattern at the center of the droplet shown as in Fig. 2(f). The experiment is performed with the same sample at the same time; thus experimental conditions such as concentration, temperature, and humidity are the same. Note that the patterns are locally different. At the edge, we observe clusters of crystals, much like in Fig. 2(e), while we observe a needle-like pattern nearby. In the following, we focus on pattern formation at the center of the initial droplet using solutions containing latex particles.

**Figure 3:**
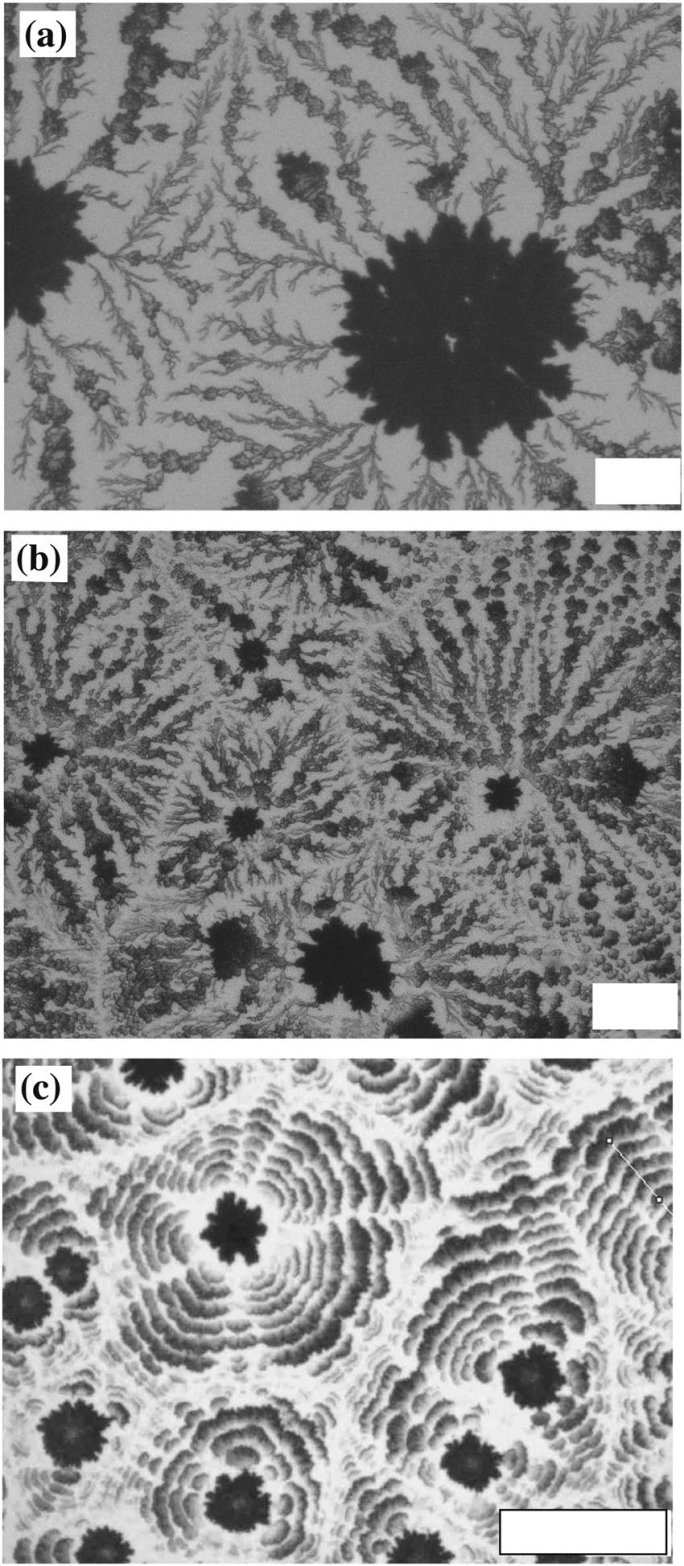
Macroscopic patterns at fixed *ϕ*_0_ = 2.8 wt% when the temperature and humidity are (**a**) 22 °C and 67%, (**b**) 22 °C and 50%, and (**c**) 22 °C and 20 ± 4%. The evaporation rates are (**a**) 0.0018 mm^3^/s, (**b**) 0.0035 mm^3^/s, and (**c**) 0.0056 mm^3^/s. We observe a dendritic pattern [(**a**) and (**b**)] for smaller *β* and concentric circles [(**c**)] for larger *β*. Scale bar corresponds to 0.1 mm.

We investigate the dependence of *β* on macroscopic crystal patterns with changing humidity. Figure 3(a–c) shows how the crystal patterns vary with initial concentration *ϕ*_0_ = 2.8 wt% at different humidities; (a) 67%, (b) 50% and (c) 20%. It is clear that *β* becomes larger with decreasing humidity. Interestingly, we observe a dendritic (DD) pattern at *β* = 0.0018 mm^3^/s and 0.0035 mm^3^/s as shown in Fig. 3(a) and (b), while a CC pattern is formed when *β* = 0.0056 mm^3^/s, as shown in Fig. 3(c). Here we classify a pattern as CC if the pattern has a periodic structure in the radial direction, and is laterally isotropic or periodic. On the other hand, the DD pattern is radially connected and random in the lateral direction. We also find that the size of the core in dendritic patterns is larger for smaller *β*. This points to the central role played by *β* in pattern formation.

**Figure 4:**
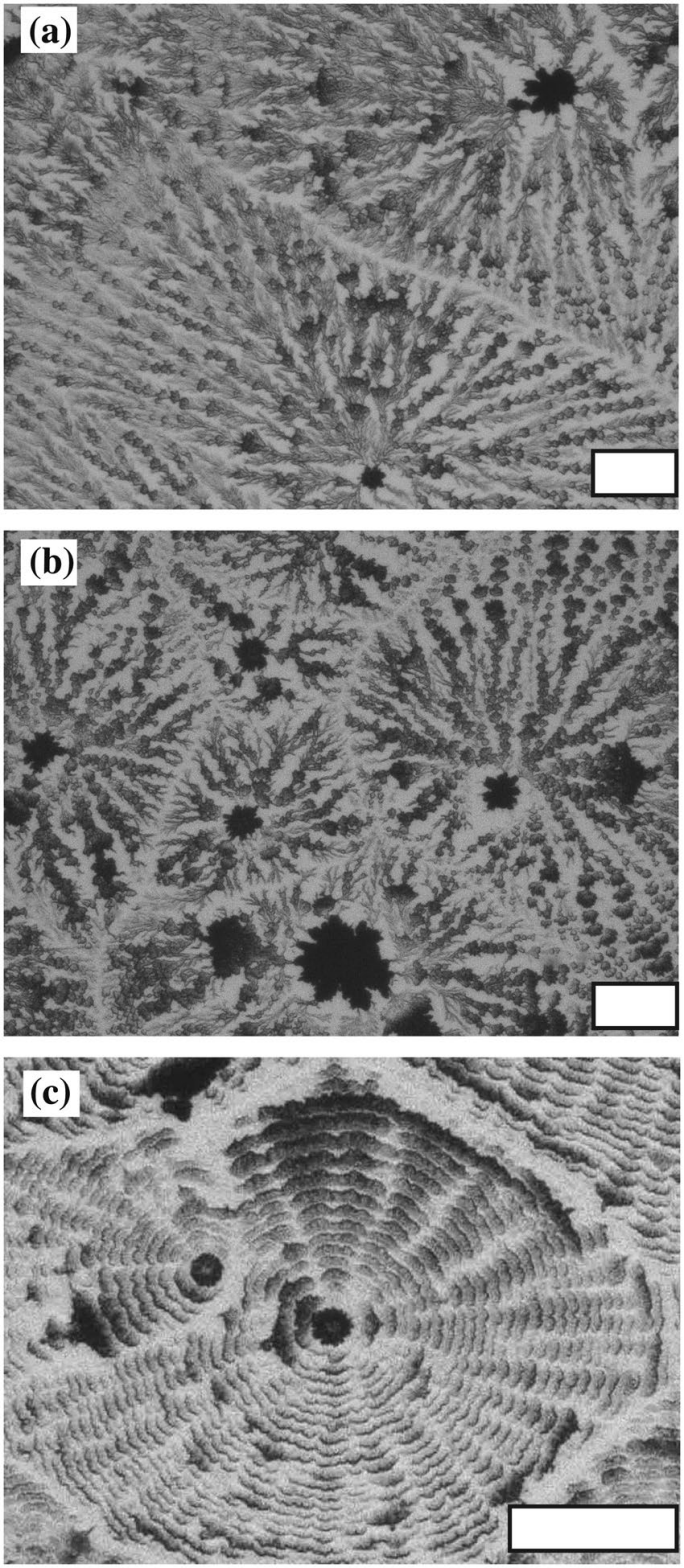
Macroscopic patterns at fixed *β* = 0.0035 mm^3^/s (22 °C, 50%) at (**a**) *ϕ*_0_ = 8.4 wt%, (**b**) *ϕ*_0_ = 2.8 wt% and (**c**) *ϕ*_0_ = 0.47 wt%. We observe both dendritic and concentric circles, despite *β* being constant. This means that *ϕ*_0_ is also an important parameter, not just *β*. Scale bar corresponds to 0.1 mm.

We also investigate the dependence of pattern formation on *ϕ*_0_ at fixed *β*. We performed evaporation experiments with different *ϕ*_0_ at the same time. Figure 4 shows the pattern formed for *β* = 0.0035 mm^3^/s with different *ϕ*_0_; (a) 8.4 wt%, (b) 2.8 wt%, and (c) 0.47wt%. At *ϕ*_0_ = 8.4 wt% and 2.8 wt%, a DD pattern is formed [Fig. 4(a) and (b)], while a CC pattern is observed for *ϕ*_0_ = 0.47 wt% [Fig. 4(c)]. This reveals that the initial concentration is also important for macroscopic pattern formation, not just the evaporation rate.

**Figure 5:**
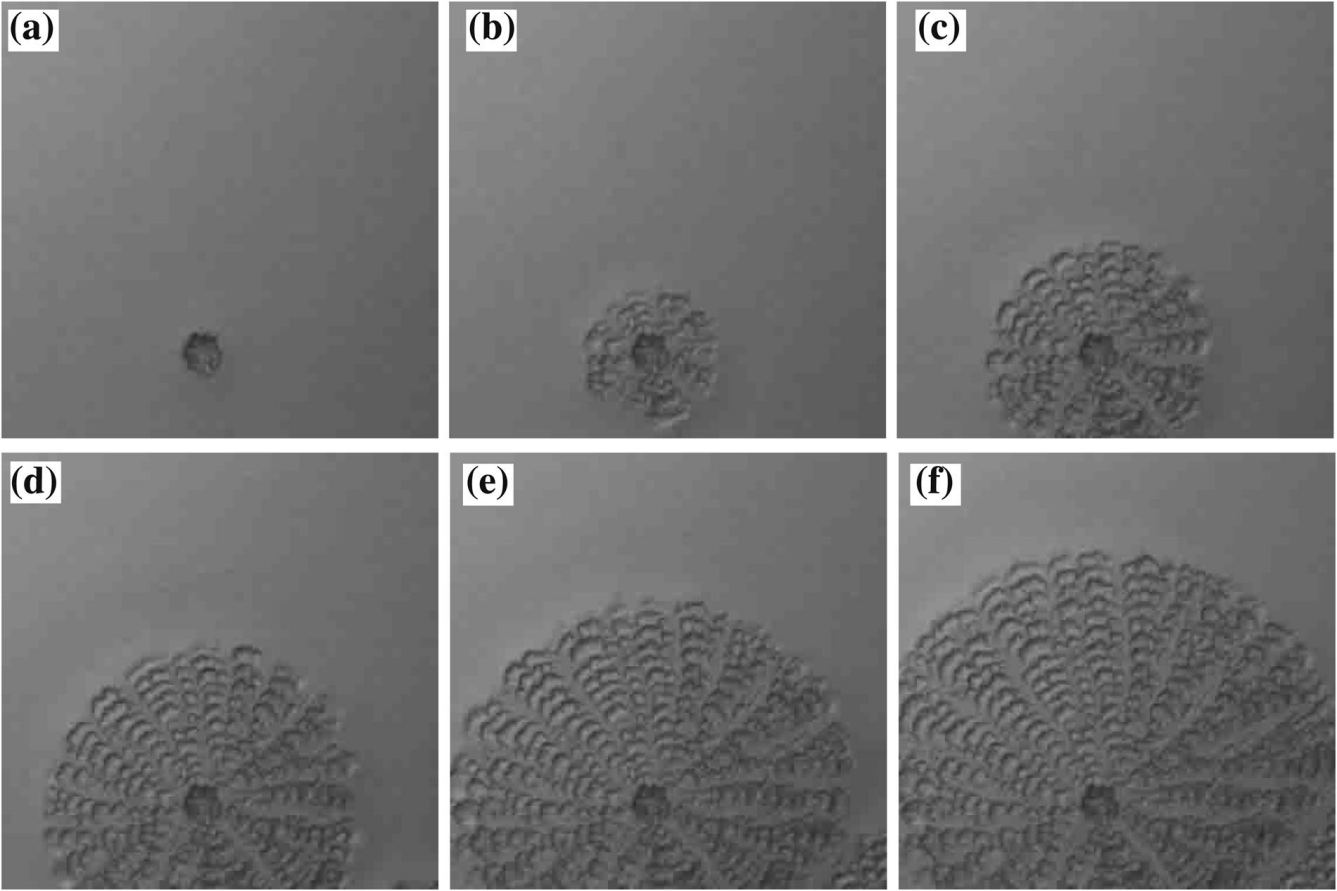
Time evolution of the CC pattern with *ϕ*_0_ = 0.47 wt% and *β* = 0.0053 mm^3^/s. (**a**) is the image at *t* = 3510 s after the experiment starts. The interval between images is 3.0 s. The size of the image is 1 mm × 1 mm.

**Figure 6:**
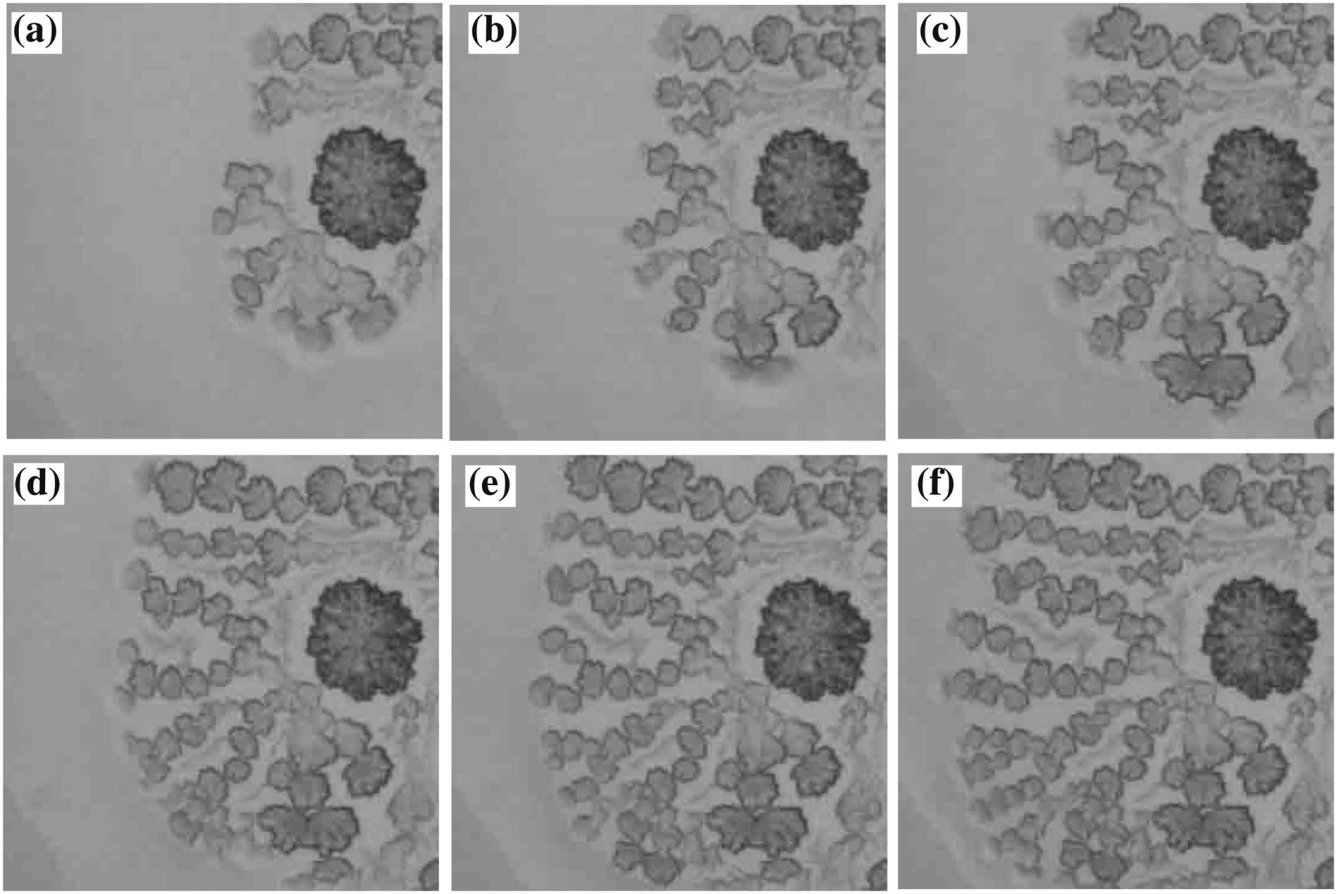
Time evolution of the DD pattern with *ϕ*_0_ = 8.4 wt% and *β* = 0.0050 mm^3^/s. (**a**) is the image at *t* = 3749 s after the experiment starts. An interval between the images is 4.0 s. The size of the image is 1 mm × 1 mm.

We go on to investigate the dynamics of crystal pattern formation. Figures 5 and 6 show the time evolution of the CC pattern and the DD pattern, respectively. We find that crystal nucleation occurs around the center of the droplet; the pattern then spreads radially from the nucleated crystal. This is quite different from CC pattern formation by stick-slip evaporation dynamics^20^. We measure the front of the pattern as a function of time and find that the pattern spreads linearly. We find that the velocity of the spread of the CC pattern is 0.015 mm/s, while that of the DD pattern is 0.0078 mm/s i.e. the CC pattern spreads approximately 2 times faster even though *β* is almost same.

**Figure 7:**
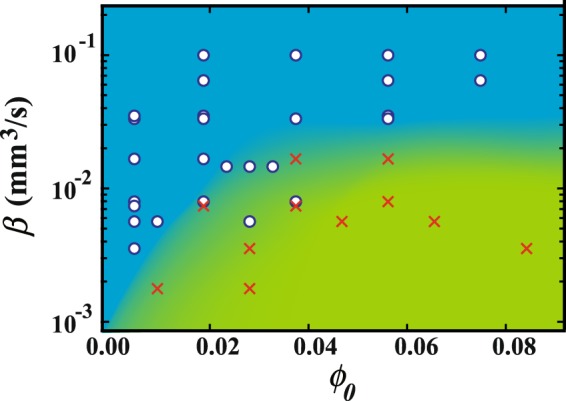
Diagram of CC and DD patterns as a function of *β* and *ϕ*_0_. Open circles and crosses correspond to CC and DD patterns, respectively. Near the boundary, we observe both patterns or an intermediate pattern which is radially periodic but laterally random.

We obtain a diagram of CC and DD patterns as functions of *β* and *ϕ*_0_ as shown in Fig. 7. Circles and crosses correspond to CC and DD patterns, respectively. We perform the same experiment for each point at least 3 times. We find that the CC pattern is observed for larger *β* and smaller *ϕ*_0_, while a DD pattern is observed for smaller *β* and larger *ϕ*_0_. Near the boundary, we observe both, or an intermediate pattern which is radially periodic but laterally random. Here we note that we changed temperature in the range between 20 °C and 60 °C. Since the temperature strongly depends on the dynamics of the crystallization, we cannot rule out the temperature effects. However, the results shown as in Figs 3 and 4 suggest that the transition between the two patterns can occur even though the temperature is same.

**Figure 8:**
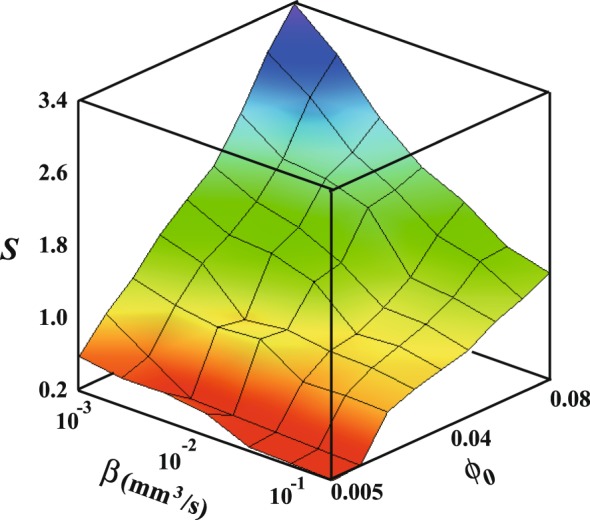
Surface plot of supersaturation *S* as a function of *ϕ*_0_ and *β*. *S* is the mean value over the droplet at *t* = *t*_*x*_. *S* between the measured points is interpolated linearly. Color corresponds to the value of *S*.

In order to investigate the effect of *ϕ*_0_ and *β*, we estimate a supersaturation $S={\varphi }_{x}/{\varphi }_{s}$ when the crystallization occurs. *ϕ*_*x*_ and *ϕ*_*s*_ are the concentration at *t* = *t*_*x*_ and the saturation concentration, respectively. *ϕ*_*x*_ is simply calculated as ${\varphi }_{x}={\varphi }_{0}{V}_{0}/{V}_{x}$, where *V*_*x*_ is the volume at *t* = *t*_*x*_. *S* is the mean value over the droplet. Here, we find that *S* dramatically increases with smaller *β* and larger *ϕ*_0_ shown as in Fig. 8. Comparing with the morphology diagram, *S* seems to be closely related to pattern formation. We also estimate the Péclet number *Pe* = *RU*/*D* for inhomogeneities in the concentration, where *R*, *U* and *D* are the radius, typical velocity and diffusion constant. The Péclet number represents the ratio of advection to diffusion. *U* can be estimated as *β*/*A*(*t*) where *A*(*t*) is the square of the cap of the droplet^25^. We use the *D* of water (1.0 × 10^−9^ m^2^/s), which is of similar order to the diffusion constant of each ion. We obtain $Pe\sim 160$ for *β* = 10^−1^ mm^3^/s and 1.6 for *β* = 10^−3^ mm^3^/s. This means that the concentration becomes high near the edge due to advection when *β* is large. Thus, *S* is less than 1 for smaller *ϕ*_0_ even though crystallization occurs. In addition, *ϕ*_*s*_ should depend on the concentration of the impurity i.e. latex particles. Near the edge, the concentration of latex particles is expected to be high; this may affect *S*. Thus, the relation between *S* and the pattern should be considered to vary over space.

**Figure 9:**
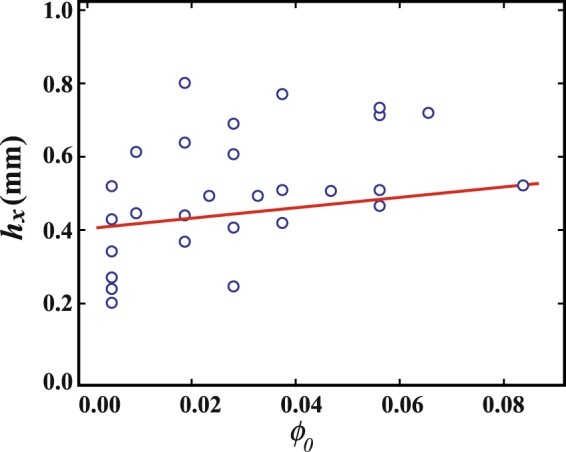
The height at *t* = *t*_*x*_ *h*(*t*_*x*_) as a function of *ϕ*_0_. *h*(*t*_*x*_) weakly depends on *ϕ*_0_, even though *S* should be large.

We then measure the height when crystallization starts, *h*(*t*_*x*_), in order to investigate the *ϕ*_0_ dependence of *S*. Figure 9 shows *h*(*t*_*x*_) as a function of *ϕ*_0_ and different *β*. We find that *h*(*t*_*x*_) weakly depends on *ϕ*_0_. Interestingly, this suggests that crystallization occurs for similar droplet shape rather than the concentration at *t*_*x*_. It is consistent that *S* is larger with the larger *ϕ*_0_. Considering both Peclet number and *h*(*t*_*x*_), the concentration in the droplet should be high and less inhomogeneous for smaller *β* and larger *ϕ*_0_, when the DD pattern is formed. That is, the concentration at the center at *t* = *t*_*x*_ should be high for the smaller *β* and the larger *ϕ*_0_, while it should be low for large *β* and small *ϕ*_0_.

**Figure 10:**
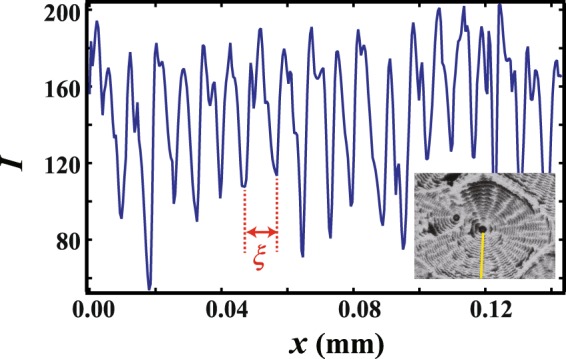
Intensity *I* over the cross section through the solid line. (inset) A concentric pattern obtained with *ϕ*_0_ = 0.47 wt% and *β* = 0.0035 mm^3^/s.

**Figure 11:**
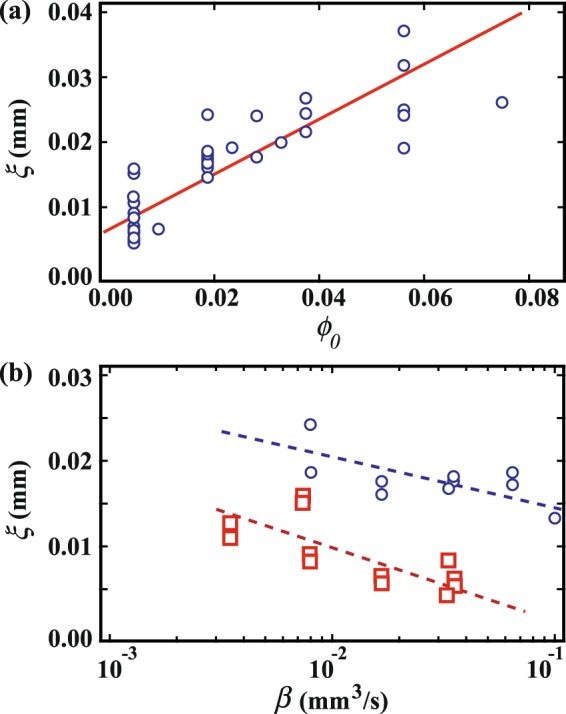
(**a**) *ξ* as a function of *ϕ*_0_. (**b**) *ξ* as a function of *β* at fixed *ϕ*_0_. Circles and squares correspond to *ξ* at *ϕ*_0_ = 1.9 wt% and 0.47 wt%, respectively. It is found that *ξ* increases with increasing *ϕ*_0_ or with decreasing *β*.

Next, we go on to investigate the period *ξ* of the concentric circles in the radial direction by analyzing the images. As an example of image analysis, we show the intensity *I* over the cross section (yellow line) in the inset [Fig. 10]. Note that *I* changes periodically; we obtain *ξ* as the average distance between the main neighboring peaks. *ξ* is averaged over 4 different lines for each set of concentric circles. We show *ξ* as a function of *ϕ*_0_ in Fig. 11(a) and *ξ* as a function of *β* at fixed *ϕ*_0_ in Fig. 11(b). It is found that *ξ* increases with increasing *ϕ*_0_ or with decreasing *β*. This suggests that *ξ* increases as we approach the region preferring DD patterns. From the point of view of morphology, concentric circles are distinct from radial patterns like the dendritic. However, we could not find a discontinuous transition between the two patterns in our experiment.

**Figure 12:**
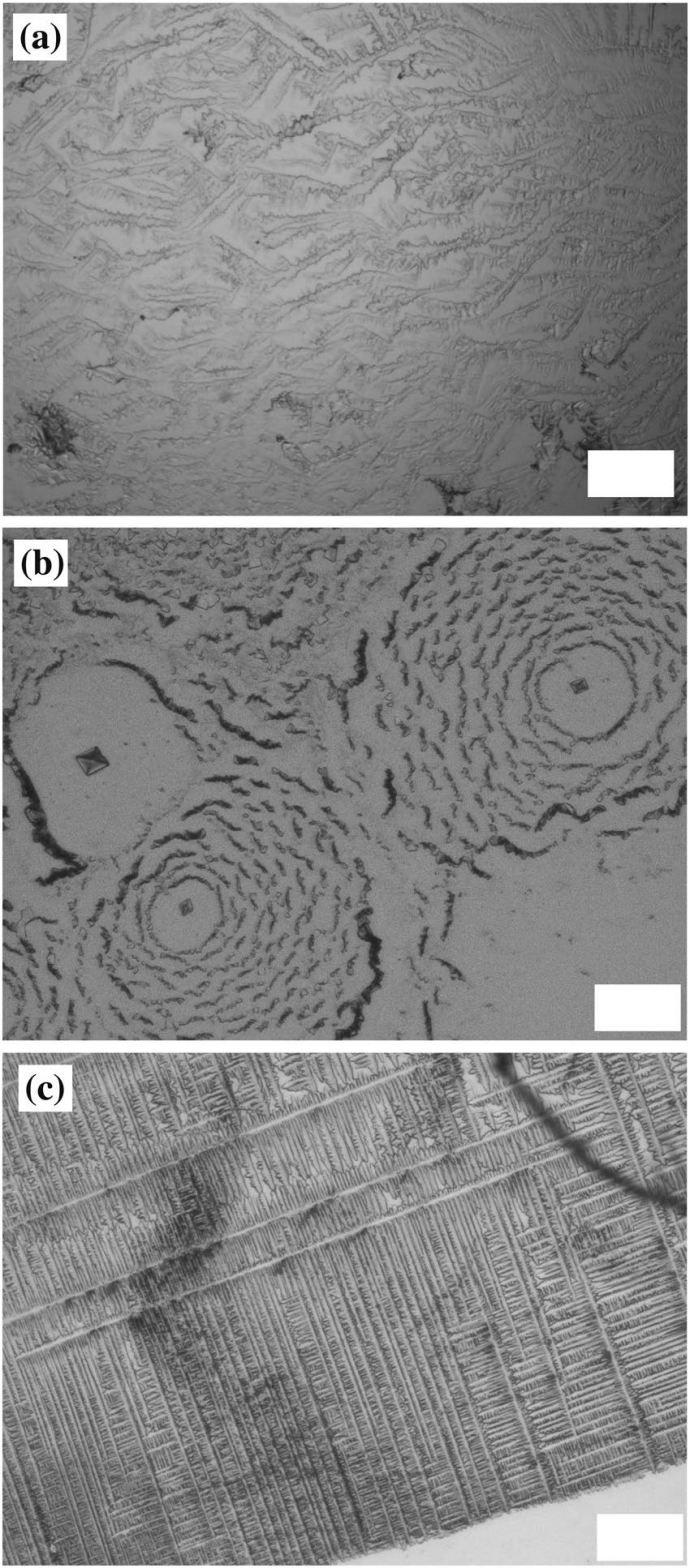
(**a**) DD pattern in NaCl with *ϕ*_0_ = 0.1 wt% at 24 °C and Rh = 60%. (**b**) CC pattern in NaCl with *ϕ*_0_ = 0.1 wt% at 18 °C and Rh = 40%. (**c**) Lattice pattern in aluminium potassium sulfate dodecahydrate solution with *ϕ*_0_ = 0.3 wt% at 18 °C and Rh = 40%.s. Scale bar corresponds to 0.1 mm.

We also examine the crystal pattern using other solutions with pinning at the edge of the droplet. Figure 12 shows the crystal pattern of a NaCl solution with *ϕ*_0_ = 0.1 wt% at (a) 24 °C and Rh = 60% and (b) 18 °C and Rh = 40%. We observe DD and CC patterns in NaCl as well as in NaHCO_3_. We note that NaCl forms a monocrystal at the center of the droplet when the edge is not pinned by latex particles^25^. A lattice pattern is obtained in aluminium potassium sulfate dodecahydrate solution with *ϕ*_0_ = 0.3 wt% at 18 °C and Rh = 40%. We thus note that various macroscopic patterns can be obtained by pinning the edge of the droplet.

Finally, we discuss the effect of dewetting from the substrate. As we showed above in Fig. 2, CC or DD patterns are formed at the center when the solution macroscopically wets the substrate. It was reported that a DD pattern is formed by dewetting in a colloidal suspension^29^. Although the crystallization is different in nature from the aggregation of the colloidal particles, the effect of dewetting seems crucial for pattern formation. Thus, we need to investigate the relationship between local concentration and the local dewetting; a challenge for the near future might be to perform an evaporation experiment with changing substrate affinity.

## Summary

In summary, we investigate macroscopic crystal patterns formed by evaporation of solution droplets. We put latex particles in the solution such that the edge of the droplet is pinned by the coffee ring effect. Firstly, we show that the concentric circles (CC) and the dendritic (DD) pattern are observed when the edge is pinned. Our systematic study shows that the CC pattern is formed for larger evaporation rate and lower initial concentration, while the DD pattern is formed for lower evaporation rate and larger initial concentration. We also suggest that a CC pattern is favored when the local concentration is low at the center; on the other hand, a DD pattern is formed if the local concentration is large. We firmly believe that this investigation will stimulate further studies to control macroscopic crystal patterns and understand crystallization mechanisms.

## Materials and Methods

We used sodium bicarbonate (NaHCO_3_) with 99% purity purchased from Wako Chemical Industries, Ltd. The saturation concentration of NaHCO_3_ solution is 8.7 wt%. We put a 20 mm^3^ droplet of the solution on a cover glass; recrystallization occurs on evaporation. For looking at macroscopic patterns with a combined coffee ring effect, polystyrene latex particles (Magsphere Co.) with 0.052 *μ*m diameter was mixed into the solution with a volume fraction 0.01%. The sample temperature was controlled with a transparent hot stage (Blast Co.) and the humidity was measured for every experiment. We observed the macroscopic crystal patterns using an optical microscope (Nikon ECSROPSE-TS100). The time evolution of the droplet size is observed with a digital camera (Panasonic, HC-W570M). We also performed similar experiments with sodium chloride (NaCl) and aluminum potassium sulfate dodecahydrate with 99% purity purchased from Wako Chemical Industries, Ltd.

## Electronic supplementary material

Crystallization dynamics during evaporation

Information of supplementary video

## References

[1] Kelton, K. & Greer, A. L. *Nucleation in condensed matter: applications in materials and biology*, vol. 15 (Elsevier, 2010).

[2] Debenedetti, P. G. *Metastable Liquids* (Princeton Univ. Press, Princeton, 1997).

[3] Taber, S. The growth of crystals under external pressure. *American Journal of Science* 532–556 (1916).

[4] Magono C (1966). Meteorological classification of natural snow crystals. Journal of the Faculty of Science, Hokkaido University. Series 7, Geophysics.

[5] Quilaqueo M, Aguilera JM (2016). Crystallization of nacl by fast evaporation of water in droplets of nacl solutions. Food Research International.

[6] Gupta S, Pel L, Steiger M, Kopinga K (2015). The effect of ferrocyanide ions on sodium chloride crystallization in salt mixtures. Journal of Crystal Growth.

[7] Moncada M (2015). Nano spray-dried sodium chloride and its effects on the microbiological and sensory characteristics of surface-salted cheese crackers. Journal of dairy science.

[8] Oikawa N, Kurita R (2016). A new mechanism for dendritic pattern formation in dense systems. Sci. Rep..

[9] Kurita R (2017). Control of pattern formation during phase separation initiated by a propagated trigger. Sci. Rep..

[10] Libbrecht KG (2001). Morphogenesis on ice: The physics of snow crystals. Engineering and Science.

[11] Honjo H, Ohta S, Matsushita M (1987). Phase diagram of a growing succinonitrile crystal in supercooling-anisotropy phase space. Phys. Rev. A.

[12] Kobayashi R (1993). Modeling and numerical simulations of dendritic crystal growth. Physica D, Nonlinear Phenomena.

[13] Wu X, Wang G, Zhao L, Zeng D, Liu Z (2016). Phase field simulation of dendrite growth in binary ni–cu alloy under the applied temperature gradient. Computational Materials Science.

[14] Parisse F, Allain C (1996). Shape changes of colloidal suspension droplets during drying. Journal de Physique II.

[15] Deegan RD (2000). Contact line deposits in an evaporating drop. Phys. Rev. E.

[16] Shahidzadeh-Bonn N, Rafai S, Azouni A, Bonn D (2006). Evaporating droplets. J. Fluid Mech..

[17] Deegan RD (1997). Capillary flow as the cause of ring stains from dried liquid drops. Nature.

[18] Man X, Doi M (2016). Ring to mountain transition in deposition pattern of drying droplets. Phys. Rev. Lett..

[19] Marin AG, Gelderblom H, Lohse D, Snoeijer JH (2011). Order-to-disorder transition in ring-shaped colloidal stains. Phys. Rev. Lett..

[20] Chen Y-J, Suzuki K, Mahara H, Yoshikawa K, Yamaguchi T (2013). Self-organized archimedean spiral pattern: Regular bundling of fullerene through solvent evaporation. Appl. Phys. Lett..

[21] Rodriguez-Navarro C, Doehne E (1999). Salt weathering: influence of evaporation rate, supersaturation and crystallization pattern. Earth surface processes and landforms.

[22] Ito M, Izui M, Yamazaki Y, Matsushita M (2003). Morphological diversity in crystal growth of l-ascorbic acid dissolved in methanol. J. Phys. Soc. Jpn..

[23] Yakhno TA (2011). Complex pattern formation in sessile droplets of protein-salt solutions with low protein content. what substance fabricates these patterns?. Phys. Chem..

[24] Sefiane K (2014). Patterns from drying drops. Adv. Colloid Interface Sci..

[25] Shahidzadeh N, Schut MF, Desarnaud J, Prat M, Bonn D (2015). Salt stains from evaporating droplets. Sci. Rep..

[26] Shahidzadeh-Bonn N, Rafa S, Bonn D, Wegdam G (2008). Salt crystallization during evaporation: impact of interfacial properties. Langmuir.

[27] Vázquez P (2015). Infrared thermography monitoring of the nacl crystallisation process. Infrared Phys. Technol.

[28] Soulie V (2015). The evaporation behavior of sessile droplets from aqueous saline solutions. Phys. Chem. Chem. Phys..

[29] Habibi M, Moller P, Fall A, Rafai S, Bonn D (2012). Pattern formation by dewetting and evaporating sedimenting suspensions. Soft Matter.

