# Supplementary material for: Emergence of different crystal morphologies using the coffee ring effect
Source: Sci Rep. 2018 Aug 21;8:12503. doi: 10.1038/s41598-018-30879-8 (PMC6104097; doi:10.1038/s41598-018-30879-8)
Supplement: Supplementary file 2 — Information of supplementary video [file 41598_2018_30879_MOESM2_ESM.pdf]

Supplementary

TITLE: Emergence of different crystal morphologies using the coffee ring effect.

Authors: Kouki Morinaga, Noriko Oikawa, Rei Kurita

(Smovie1) Crystallization dynamics in a sessile droplet between t = 4665 s and t = 5848 s. A left droplet is a 0.47 wt¥% droplet including the latex particles, on the other hand, a right droplet is is a 0.47 wt¥% droplet without the latex particles. This experiment is same as Fig. 2 in the main manuscript.
